# How to promote grass roots medical treatment under China's graded diagnosis and treatment policy? — From the perspective of customer value theory

**DOI:** 10.3389/fpubh.2022.994644

**Published:** 2022-11-29

**Authors:** Wanqiong Tao, Xiangrui Chen, Shuyu Gan

**Affiliations:** ^1^Collaborative Innovation Center of Statistical Data Engineering Technology and Application, Zhejiang Gongshang University, Hangzhou, China; ^2^School of Management Engineering and E-business, Zhejiang Gongshang University, Hangzhou, China; ^3^School of Management, Zhejiang University, Hangzhou, China; ^4^Zhejiang Geely Holding Group Co., Ltd., Hangzhou, China; ^5^School of Accounting, Zhejiang Gongshang University, Hangzhou, China

**Keywords:** grass-roots medical treatment rate, difference-in-difference model, customer value theory, primary medical institutions, graded diagnosis and treatment policy

## Abstract

**Objectives:**

Based on the background of the implementation of graded diagnosis and treatment policy (GDT) in China, this paper studied the service optimization of primary medical institutions from the perspective of the primary medical treatment rate.

**Methods:**

First, the difference-in-difference (DID) empirical strategy is used to analyze the impact of the implementation of the GDT on the improvement of the primary medical attendance rate, and the parallel trend test and the placebo test were used to test the robustness to illustrate the necessity of policy implementation. Second, combined with customer value theory, this paper used a logistic regression model to study the impact of customer value perception on grassroots medical care.

**Results:**

The implementation of the GDT can indeed improve the rate of primary care (*p* = 0.028), but the impact is relatively small (0.042). From the perspective of value perception hierarchy, emotional value perception (*p* < 0.05) is the key factor that affects the behavior of grassroots medical treatment at this stage.

**Conclusion:**

This paper fills the gaps in existing research, including empirical research on the grassroots medical treatment behavior of the masses under GDT and the impact of value perception on grassroots medical behavior. Furthermore, the recommendations are made for primary care institutions based on the results of the analysis, which promote the advancement of primary care services in China.

## Introduction

The graded medical system is an important strategic measure to integrate medical resources and optimize medical patterns in China. Primary medical institutions are an important part of China's medical system. In the post-epidemic era, the potential risk of epidemic outbreaks and the frequent occurrence of public health security events require hospitals at or above the second level to take on key tasks. Therefore, how to reasonably allocate medical resources, consider the monitoring and prevention of epidemic situations, and meet people's basic medical needs has become the focus of the topic. The full realization of grassroots medical services to improve people's medical services and ensure adequate resources for epidemic prevention is of great significance. Normalized epidemic prevention pressure cannot be relieved for the time being, and the growing multi-level health consultation and daily healthcare needs of the masses need to be met urgently. Hence, it is essential to optimize the services of grassroots medical institutions and guide the masses to seek medical treatment reasonably.

It is an important part of China's new medical reform to strengthen the construction of grassroots medical institutions and build a graded diagnosis and treatment (GDT) system for basic diseases. In 2009, *the Opinions of the State Council of the CPC Central Committee on Deepening the Reform of Medical and Health System* proposed to improve the new urban medical and health service system based on grassroots health services and gradually realize the GDT. In 2013, after *Guiding Opinions of the General Office of the State Council on Promoting the Construction of a Graded Diagnosis and Treatment System*, the Reform emphasized: “improving the GDT service system by focusing on strengthening the grassroots,” several provinces and municipalities have successively carried out the pilot work of the regional GDT system. The promulgation *of The Guiding Opinions of the General Office of the State Council on Promoting the Construction of Graded Diagnosis and Treatment System* in 2015 marks the beginning of the comprehensive implementation of GDT in China.

Unlike the compulsory primary care policy of the UK and Germany (1, 2), a non-rigid GDT system guided by policies such as administrative leadership and differentiated medical insurance reimbursement has been adopted in China, whereby the people have more discretion in their choice of treatment (3). Grassroots medical institutions provide diagnosis and treatment consulting services to the masses is an important part of the GDT system.

Graded diagnosis and treatment is a division of labor arrangement between different levels of health institutions based on the vertical integration of healthcare and for the purpose of seeking accessible and effective medical services for the people (4). At present, many scholars believe that the implementation of GDT in China is ineffective and difficult. Mainstream scholars summarize the reasons for the difficulty in forming a GDT order in two aspects. At the level of macro-policies and systems, there are designs that are not conducive to the implementation of GDT policies, including the graded system of medical institutions, compensation mechanisms, and medical insurance reimbursement systems. Yao (5) proposed that the “system embeddedness” of GDT due to historical reasons was not good, and the market-oriented characteristics of the medical industry in China led to resistance to the implementation of GDT (1); Lu et al. (6) proposed that the lack of medical service ability in primary medical institutions is the reason why the graded diagnosis and treatment order is difficult to form; Xiao et al. (7) proposed that the current medical system had the problem of insufficient input of medical resources and uneven allocation of medical resources, and the compensation mechanism of public hospitals was unreasonable. In the micro-level of the graded medical system, the lack of equipment resources, talent vacancy, poor publicity, and other reasons lead to resistance to the development of GDT. Yang et al. (8) highlighted that the concentration of medical resources in large hospitals led to inadequate health resources at the grassroots level, which increased the disadvantage of grassroots medical institutions in the medical system; Li et al. (9) stated that high-quality primary healthcare services took grassroots general practitioners as “gatekeepers” and that the talent shortage would limit the sustainable development of the primary healthcare system; Ma et al. (10) from the perspective of key practitioners, found that the publicity was inadequate, leading to inadequate understanding of the GDTs system by doctors and patients.

In China, the research on the choice of medical institutions for the masses mainly focuses on the differences between primary medical institutions and large hospitals and the relationship between these differences and the behavior of the masses. According to Liu et al.'s analysis of the 2015–2020 Health Statistics Yearbook of China, the growth rate of visits, the utilization rate of diagnostic beds, and the growth rate of health technical personnel showed that the development of primary medical institutions was less than that of second- and third-class medical institutions. An imbalance in the development of medical institutions led to negative guidance to the grassroots medical treatment behaviors of the masses (11). Based on data from Zhongshan City, Guangdong Province, Yue et al. found that there were differences in the accessibility of facilities among different institutions, which affected the medical care of people (12). Wang et al. (13) found that the main reason for the masses to seek medical treatment in large hospitals is the level of diagnosis and treatment, and the convenience of medical treatment and good service attitude of medical staff are important factors affecting the masses to seek medical treatment at the grassroots level. Based on Shanghai's data, Ruan et al. (14) identified the inconsistency between the masses' medical treatment behavior and the GDT, driven by the relevant reasons of the masses (habits, trust, and knowledge), physicians (conservative priority and risk avoidance), and systems (accessibility and operability). According to the research of Wang Jin and Zhu Yimin (15), the imperfect basic drug system and residents' cognitive bias restrict the implementation of primary consultation.

Existing studies have evaluated the effectiveness of GDT and made some attributions in terms of policy system, publicity, personnel allocation, etc. These studies are mainly based on descriptive analysis, and a small number of studies using quantitative analysis have not paid attention to grassroots medical treatment. In addition, in the existing research on grassroots medical behavior, the discussion on the masses is limited, and it is seldom analyzed from the perspective of the value perception of grassroots medical institutions.

Promoting grassroots medical care is an important goal of the implementation of GDT and relates to the daily lives of hundreds of millions of people. However, according to the data disclosed in the China Health Statistics Yearbook, the average annual growth rate of hospital visits was around 5% from 2015 to 2019, while the average annual growth rate of visits in primary medical institutions is only 1%. It is worth discussing how to optimize the effect of GDT to promote the effectiveness of primary care. The competitiveness of an economic subject depends on the customer's recognition of the product or service value. Therefore, to increase the rate of primary care, it is of great importance to fully understand the impact of customer value perception hierarchy on primary care behavior.

Due to insufficient existing research, this paper conducted a quantitative analysis of primary care visits. We hoped to address the following questions: (1) Does the implementation of GDT have an impact on primary care visits? (2) Does the public's perception of customer value affect grassroots medical treatment behavior? The research ideas of this paper are as follows. First, based on the data of some cities in the 2013–2016 China Statistical Yearbook, the DID model is used to quantify the impact of the graded treatment policy on grassroots medical attendance. Second, using the theoretical model of customer value composition, from the three dimensions of “function value, emotional value, and social value,” this paper analyzed the impact of perception degree on the grassroots medical treatment behavior of the masses and realized the positioning of the level of customer value perception. Moreover, it put forward reasonable and effective suggestions for the optimization of primary medical institutions' services under the background of the implementation of GDT.

The contributions of this paper are mainly reflected in the following two aspects (1): Existing research on GDT in China is mainly based on descriptive analysis. Although a small amount of literature quantitatively analyzes the implementation of the GDT system in China, there is almost no research on the main body of primary medical institutions. This paper takes the primary-level medical treatment rate as the main observation index and quantitatively analyzes the impact of the GDT pilot on the operation of primary medical institutions, which fills the gap in this research field to a certain extent (2). This paper innovatively starts from the theory of customer value, examines the impact of value perception on the grassroots medical treatment behavior of the masses, and provides guidance for optimizing grassroots medical services.

## Theoretical foundation

Since the 1990s, customer value has been closely concerned by researchers and entrepreneurs, and it has a profound impact on the decision-making of many economic subjects. The mainstream view is that customer value is a subjective perception that consumers weigh gains against losses when they receive services from economic agents (16). Based on Sheth's customer value theory (17), Sweeney and Soutar divide customer value into the following three dimensions: functional value, emotional value, and social value (18). Customer values perception satisfies the following formula:


(1) $$\begin{array}{l} CVP\;=\;f\;\left(FVP,\;EVP,\;SVP\right)\;=\;f\;\left(CPI,\;CPE\right) \end{array}$$

where CVP means customer value perception, FVP means functional value perception, EVP means emotional value perception, SVP means social value perception, CPI means customer perception, and CPE means customer perception. Existing studies generally believed that there is a complex non-linear relationship between the elements, showing the characteristics of hierarchy, flow, and so on (19, 20). Hierarchy is embodied in the fact that each value dimension is independent, interrelated, and contributes to selection progressively. Functional value is the basic factor affecting consumer choice. Only when the perceived value of lower-level reaches a certain threshold, the demand for higher-level value will appear and affect the behavior of the masses (21, 22). Liquidity is embodied in that the same customer has different value perceptions in different dimensions. This paper will orient the level of value perception of the masses in China on primary medical services and explore the impact of the above values on the behavior of the masses.

### Effectiveness analysis of GDT Policy to promote the primary care

#### Method

Based on the yearbook data of many cities in China, the difference-in-difference model (DID) is used in this paper to analyze the effectiveness of GDT in improving grassroots medical behavior. As a regional policy, the pilot program of GDT can be regarded as an exogenous variable. In this paper, the DID empirical strategy is used to test whether the GDT increases grassroots medical treatment behavior by analyzing the differences between the experimental group and the control group. In August 2014, the *Implementation Plan of Graded Diagnosis and Treatment Pilot Work in Zhejiang Province* (the “Plan”) was released, including the first batch of GDT pilots in Zhejiang Province launched in several counties (districts) under the jurisdiction of Ningbo City and Wenzhou City. After the issuance of a notice promoting the pilot work of GDT in August 2016, the pilot program of GDT in 266 prefecture-level cities nationwide, including Guangzhou City and Wuhan City, has been officially launched. Different regions have different times to implement the GDT, so the DID model is applicable.

#### Data collection

There are differences in the official implementation of GDT pilots in various regions of China. As early as the end of the third-quarter of 2014, Zhejiang Province is one of the earliest regions to carry out the GDT pilot program in China, and it is a strong representative as a sample. Therefore, the first batch of pilot cities in Zhejiang province was selected as the experimental group. In the control group, the three cities of Suzhou, Guangzhou, and Wuhan were selected, and the sample involved eastern, southern, and central China, which were widely distributed and strongly representative. The data required for the experiment are obtained from the statistical yearbooks and health yearbooks of each city from 2013 to 2016.

To test whether the GDT system can effectively promote grassroots medical treatment behavior, the grassroots medical treatment rate is used to represent the grassroots medical treatment behavior of the masses. The formula for calculating the grassroots medical treatment rate in this paper is as follows:


(2) $$\begin{array}{l} S\;=\;\left(OEV-\;HOEV\right)/OEV \end{array}$$

where *OEV* refers to the number of patients treated in the city during the year, and *HOEV* refers to the number of patients treated in general hospitals at or above the county level during the year. Overall, medical institutions classified and disclosed in China's statistical yearbook, hospitals, and primary medical institutions account for an absolute proportion, and professional public health institutions account for a very small proportion, so the above formula is calculated.

We assumed that the consumption level, the degree of local economic development, and the allocation of medical resources may affect the experimental results by affecting the public's health concept, diagnosis, and treatment choices. Therefore, some control variables were set in these aspects. Urban per capita consumption expenditures (urban population meets total household daily consumption expenditure) and rural per capita consumption expenditure (rural population meets household daily consumption total expenditure) are set to control the difference in consumption levels between regions. GDP per capita is set as a random variable to control for economic disparity. The number of health technicians per 1,000 people (the number of professionals currently engaged in health technology work among all practitioners paid by health institutions per 1,000 people) is set as a random variable to control for differences in medical resources.

#### Model design

The following DID model is designed to explore the influence mechanism of the graded diagnosis and treatment policy on the primary care rate.


(3) $$\begin{array}{l} S_{it}=\theta_{0}+\theta_{1}time_{t}*treated_{i}+\theta_{2}X\;+\varepsilon_{i} \end{array}$$

Where *S*_*it*_ indicates the primary consultation rate in the *i* area in the *t* year. The dummy variable *treated*_*i*_ is a dummy variable that indicates whether or not a pilot area is for graded treatment. The value of *treated*_*i*_ in the pilot areas (Wenzhou and Ningbo) is 1, and that in the control areas (Guangzhou and Wuhan) is 0. The dummy variable *time*_*t*_, which represents a dummy variable that indicates whether the pilot graded diagnosis and *time*_*t*_ has been conducted in that year, due to the implementation of the GDT in Wenzhou and Ningbo in the third-quarter of 2014. Considering the lag effect of policy implementation, 2014 and the time before 2014 are regarded as the pre-implementation stage of the policy. The value of *time*_*t*_ is 0, 2015, and the time after 2015 is the post-implementation stage of the policy, and *time*_*t*_ is 1. *time*_t_^*^treated_*i*_ represents the interaction between the virtual variable and the experimental group variable during the experimental period. *X* is a set of random variables, including the per capita consumption expenditure of urban residents, per capita consumption expenditure of rural residents, per thousand technicians, and per capita GDP. ε_*i*_ is a random error term.

The parameter of interest in this paper is θ_1_, if θ_1_ is significantly positive, it can be inferred that the GDT is effective in guiding people to see a doctor at the grassroots level. The magnitude of θ_1_ indicates the extent to which the GDT affects primary care attendance.

### Empirical analysis

The principle of DID model is to use OLS regression to test the effect quantity. The value of the interaction between treated and time is the DID double difference effect value of the study. The empirical results are shown in Table 1.

**Table 1:** Effects of GDT on primary care behavior.

**Variable**	**Grassroots treatment rate**
time_t_^*^treated*_*i*_*	0.042^*^ (2.677)
treated*_*i*_*	0.089 (1.943)
time*_*t*_*	−0.002 (−0.128)
Rural per capita consumption expenditure	0.025^*^ (−3.058)
Health technicians per thousand	−0.014 (−0.493)
Per capital gross regional product	0.002 (0.129)
constant	0.411^**^ (−20.382)
sample size	16
*R* ^2^	0.987
Adjust *R*^2^	0.976

* *p* < 0.05,

** *p* < 0.01.

#### Results

As can be seen from the second line, the coefficient of *time*${}_{t}^{*}$treated_*i*_ is 0.042 (*p* = 0.028), which is significant at the level of 5%, indicating that after the implementation of the GDT policy, the rate of grassroots medical treatment in pilot areas has increased by 4.2% compared with non-pilot areas, and the GDT policy has a positive impact on the grassroots medical treatment behavior of the masses, but the impact is limited.

In the control variables, urban per capita consumption expenditure has a significant negative impact on grassroots medical treatment (line 5, *p* = 0.000), and rural per capita consumption expenditure has a significant positive impact on grassroots medical treatment (line 6, *p* = 0.016). It can be concluded that the urban areas realized the well-off earlier, and the residents' basic living needs were satisfied, which promoted the generation of higher healthcare needs. Therefore, the higher the level of public consumption, the more likely to visit a large hospital. Due to the imbalance of medical resources, large hospitals in urban areas are concentrated, and the masses have more independent choices in medical treatment. The phenomenon of over-level medical treatment and over-diagnosis shown in the data is that the higher the urban per capita consumption expenditure, the lower the grassroots medical treatment rate. In contrast, in rural areas, the economic level is relatively low, medical resources are relatively limited, and the distribution of large hospitals is small. With the increase in the consumption level of the population, the demand for medical care will increase, and so will the behavior of people seeking medical treatment and the grassroots treatment rate.

#### Robustness

For the above analysis results, this paper used a parallel trend test and a placebo test for robustness.

(1) Parallel Trend Test

**Figure 1:**
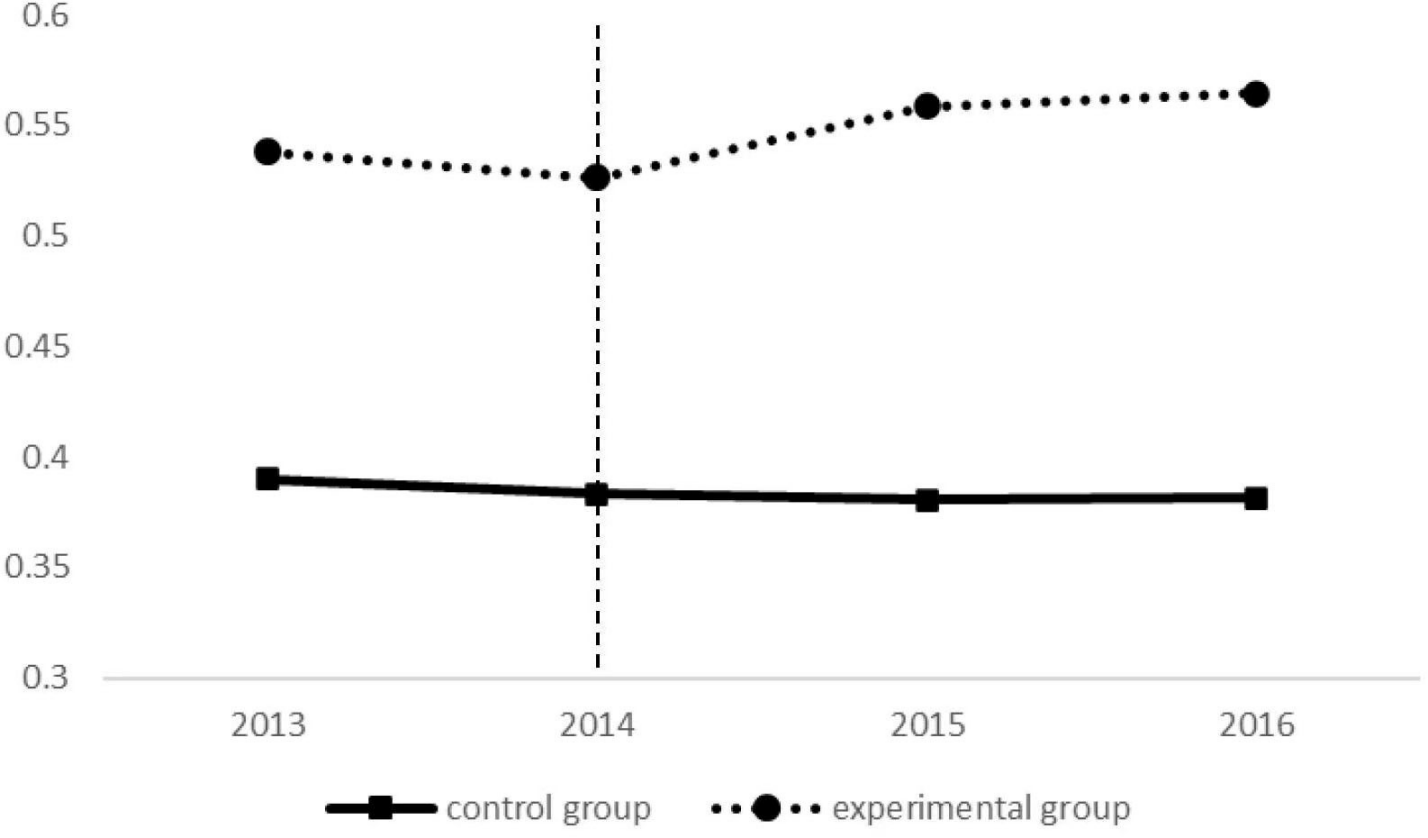
Parallel trend analysis.

In this paper, the trend of observation variables of the experimental group and control group is plotted, and the following results are obtained in Figure 1.

The trend of changes in the indicators of grassroots hospitalization rates of the experimental group and the control group before the pilot program of GDT (2013–2014) is the same. The trends of changes in the indicators of the two groups are different after the pilot program of GDT, which can better meet the parallel hypothesis trend. So, it is reasonable to use DID model to analyze the above problems.

(2) Placebo Test

According to the method in the other document 3, we setup other virtual experiment groups. The cities that have not carried out the pilot program of GDT are divided into the experimental and the control groups. If the result of DID is significant, it means that the difference in the grassroots consultation rate is not necessarily caused by the GDT but may be caused by the intrinsic characteristics existing in different regions. Wuhan, Suzhou, and Guangzhou, which have not carried out the pilot program in 2014, were selected as the experimental group, and the remaining groups were selected as the control group. As shown in Table 2, the coefficients of the treated^*^time interaction items are not significant, suggesting that the inter-group differences are not due to intrinsic regional characteristics. The research of this paper is steady.

**Table 2:** Results of placebo test.

**Variable**	**Grassroots treatment rate**		
	**Wuhan**	**Suzhou**	**Guangzhou**
treated*time	−0.038 (−1.565)	−0.047 (−1.660)	−0.037 (−1.432)
Control variable	Yes	Yes	Yes
*R* ^2^	0.961	0.955	0.975
F number	F (7, 4) = 14.163, *p* = 0.011	F (7, 4) = 12.206, *p* = 0.015	F (7, 4) = 21.911, *p* = 0.005

### Analysis of the relationship between mass value perception and medical treatment behavior under customer value theory

The results of the above empirical analysis showed that the development of GDT has a limited role in promoting grassroots medical treatment behavior. In order to improve the effectiveness of GDT, this paper analyzed the influence of different levels of value perception on grassroots medical treatment behavior by a logistic regression model.

### Method

Based on questionnaire survey data, a logistic regression model is used to study the impact of customer value perception on grassroots medical treatment behavior. In this paper, the average positive perception of each investigator in a certain dimension is used as the independent variable, and the occurrence of whether the individual chooses to seek medical care at the grassroots level is used as the dependent variable.

### Data collection

We use a random sampling method and send out questionnaires on the Internet and on-site to collect relevant data. In order to effectively control the quality of the questionnaire, at the stage of questionnaire collection, a small reward was provided at the end of the questionnaire, and it was explained in advance at the beginning of the questionnaire to improve the enthusiasm of the target object to participating in the investigation. At the same time, at the beginning of the questionnaire, bold fonts are used to explain the importance of real answers and reduce the randomness of respondents' answers. In the stage of questionnaire sorting, all the collected questionnaires are screened, and the invalid questionnaires, including those with incomplete answers and obviously random answers, are eliminated. The random questionnaires are identified by manual screening. The content of the questionnaire is divided into two parts: basic information and value perception. The basic information survey includes as follows: gender, age, place of residence, occupation, per capita monthly income of the family, health status, whether there is medical insurance, and whether the usual medical institutions are grassroots medical institutions; the value perception survey includes the following three dimensions: functional value, emotional value, and social value. Cronbach α results of all three dimensions were above 0.7 (Table 3), indicating good reliability of the questionnaire. KMO and Bartlett tests were used to validate the validity of the questionnaire. The KMO value was 0.931, and the spherical Bartlett test result was <0.01, indicating that the validity of the questionnaire was good.

**Table 3:** Reliability of the questionnaire.

**Research dimension**	**Number of topics**	**α coefficient**
Functional value	7	0.845
Emotional value	6	0.869
Social value	3	0.742

The respondents of the questionnaire are residents aged 18 and above, with clear consciousness and normal spirit. A total of 522 questionnaires were collected, and 489 were valid and complete. The validity rate of the questionnaire was 93.68%. The respondents of the questionnaire were all from pilot cities, and the proportion of the urban population was 62.7%, and the proportion of the rural population was 37.2%, which was similar to the proportion of urban and rural populations in China's Seventh Population Census (urban population proportion 63.89% and rural population proportion 36.11%), indicating that the questionnaire can reflect the grassroots medical needs of different regions. The basic information about the respondents is shown in Table 4.

**Table 4:** Statistical table of basic information of respondents.

**Variable**	**Grouping**	***N***	**Proportion**	**Variable**	**Grouping**	***N***	**%**
Sexuality	Female	278	56.8%	Monthly income per household	<2,000	91	18.6%
	Male	211	43.1%		2,001–5,000	258	52.7%
Age	<18	7	1.4%		5,001–10,000	111	22.6%
	18–27	70	14.3%		10,001–30,000	19	3.8%
	28–37	68	13.9%		>30,001	10	2.0%
	38–47	58	11.8%	Health status	Health	273	55.8%
	48–57	75	15.3%		Sub–health	194	39.6%
	58–67	166	33.9%		Indisposition	22	4.4%
	>68	45	9.2%	Medical insurance	Yes	414	84.6%
Occupation	Public institutions	47	9.6%		No	75	15.3%
	Service crew	68	13.9%	Place of residence	Rural area	307	62.7%
	Freedom workers	125	25.5%		Urban area	182	37.2%
	Worker	84	17.1%				
	Company employees	54	11.0%				
	Other occupations	111	22.6%				

### Variable measurement and survey tools

Value perception refers to the trade-off and evaluation between the attributes and efficacy of the product or service expected by the customer, as well as the outcome of the use of the product or service to help the customer achieve its objectives and the corresponding cost in a specific context (17). Value perception has the characteristics of subjectivity and variability. Environment, events, and personal experience affect people's value perception in different stages. This paper intends to explore the general mass value perception of grassroots medical institutions at this stage by setting five grades of satisfaction to quantify the value perception. If the feedback result of a factor is “very good” or “relatively good,” the applicant is considered to have a positive perception of the value under that dimension, which is recorded as 1. If the feedback is “general” or “poor” or “very bad,” the questionnaire is considered to show a non-positive perception of the value of the dimension, recorded as 0. According to China's medical grading and classification standards, “township and town health centers,” “community health centers and village clinics,” and “private clinics” belong to grassroots medical institutions. If the masses seek medical treatment in the above institutions, they shall be recognized as primary medical institutions and recorded as 1. Similarly, the masses in the “hospitals at or above the county level” is identified as non-grassroots treatment, recorded as 0. The average value of positive perception of all elements in a dimension is recorded as the positive perception value of this dimension.

### Model design and empirical analysis

To explore how value perception affects grassroots medical treatment behavior, this paper constructs a model based on grassroots medical treatment behavior:


(4) $$\begin{array}{l} Y=\beta_{0}+\;\beta_{1}X\;+\;\varepsilon \end{array}$$

where *Y* indicates whether people choose to seek treatment at the grassroots level. Independent variable *X* is the set of functional value, emotional value, and social value perception. The coefficient of concern is β_1_, which measures the effect of a certain dimension of value perception on grassroots medical behavior, and ε is a random error term. This formula is designed to explain whether the public's perception of the value of primary medical services has an impact on primary medical treatment behavior and the direction of the impact.

(1) Analysis of Value Perception in each Dimension

In this paper, the satisfaction degree of each dimension in the questionnaire is assigned and calculated, and the results are shown in Table 5.

**Table 5:** Value perception of residents.

**Dimensions**	**Factors**	***N***	**Positive perception**	***N***	**Non-positive perception**	**Average positive perception**
Functional value	Location convenience	362	74.0%	127	25.9%	71.66%
	Diagnostic accuracy	340	69.5%	149	30.4%	
	Medical technology level	352	71.9%	137	28.00%	
	Service efficiency	350	71.5%	139	28.4%	
	Instrument completeness	344	70.3%	145	29.6%	
	Rigorous operation.	345	70.5%	144	29.4%	
	Sanitary conditions	360	73.6%	129	26.3%	
Emotional value	Available communication time	349	71.3%	140	28.6%	69.97%
	Service enthusiasm	345	70.5%	144	29.4%	
	Enhancing confidence in treatment	329	67.2%	160	32.7%	
	Patience of medical staff	355	72.5%	134	27.4%	
	Personnel initiative	336	68.7%	153	31.2%	
	Privacy of diagnosis and treatment	339	69.3%	150	30.6%	
Social value	Help patients return to normal social life	357	73.0%	132	26.9%	70.07%
	Inspire patient's healthy lifestyle	354	72.3%	135	27.6%	
	Forming a “patient—doctor” community	317	64.8%	172	35.1%

(2) Analysis of the Impact of Value Perception on Grassroots Medical Behavior

In this paper, the average positive value perception within each dimension is taken as an independent variable, and the occurrence of primary care-seeking behavior is taken as the dependent variable. The logistic regression model was used. The results are shown in Table 6.

**Table 6:** Summary of logit regression analysis results.

**Variable**	**Regression coefficient**	**OR**	**95 % CI**	***P***
Functional value	−0.519	0.595	0.263–1.348	0.213
Emotional value	1.316	3.729	1.820–7.638	0.000
Social value	−0.337	0.714	0.352–1.446	0.349

Dependent variable: whether to choose primary medical service. McFadden *R*^2^: 0.022, Cox and Snell *R*^2^: 0.028, Nagelkerke *R*^2^: 0.038.

From Table 5, we can see that the average positive perception rate of the functional value dimension is 71.66%, which shows that primary medical services can provide the masses with high and sensible functional value. It is worth noting that, in this dimension, “instrument completeness” (70.3%) and “service efficiency” (70.5%) mass satisfaction is lower than the average level (71.66%). The lower satisfaction with these two factors indicates the weak ability of primary medical service, which is related to the unbalanced distribution of resources in China's medical system. The positive perception rate of emotional value (69.97%) was lower than that of functional value (71.66%). Among them, “personnel initiative” (68.7%) and “enhance confidence in treatment” (67.2%) of low satisfaction, indicating that the professional quality of grassroots medical staff needs to be strengthened. The perceived value of “privacy of diagnosis and treatment” (69.3%) was low, which may be related to the design of the diagnosis and treatment process and resource limitations. Under the social value dimension (70.07%), the most obvious problem is that the primary medical institutions cannot construct a good “patient–doctor” community (64.8%).

From Table 6, according to the second line of the form, emotional value perception has a significant impact on whether or not the masses seek medical treatment at the grassroots level (*p* < 0.05), and the regression coefficient is 1.316, indicating that the higher the mass emotional value perception of the grassroots medical institutions, the more likely they are to seek medical treatment at the grassroots level. The experimental results show that the functional value (*p* = 0.213) and social value (*p* = 0.349) have no significant impact on grassroots medical treatment behavior.

## Discussion

The general mass value perception of primary medical services is not bad, and the positive value perception distribution has a ladder-like characteristic. The proportion of positive mass value perception in the three dimensions is more than 60%. With the continuous development of the national medical system and the improvement of health supervision, the primary medical institutions have reached the value perception index of the patients and gained the majority's satisfaction and trust. Functional value, emotional value, and social value show a decreasing trend, which is matched with the demand distribution. It shows that the current services of grassroots medical institutions can scientifically meet the functional needs of the bottom, and the design is reasonable.

Emotional value perception has a positive effect on the behavior of people choosing primary medical institutions, but functional value and social value have no significant effect on them. According to the theory of demand hierarchy, after the demand of the lower level is satisfied, the demand of the higher level will appear and become the incentive factor of behavior. This study found that the level of Chinese people's perception of medical value has risen from basic functional value to higher emotional value. Over the years, China has carried out supply-side structural reform of administrative domination for medical services, and the improvement of medical institutions' treatment capacity has been able to provide the masses with fully perceived functional value. Therefore, the key factors affecting grassroots medical behavior have changed from functionality to emotion.

At present, the masses of healthcare providers in the attitude, environmental comfort, privacy, and other emotional value of the request for a higher level. Improving the perception of mass emotional value has become the growth point of the grassroots consultation rate and the breakthrough point of dredging the GDT system. In addition, functional value is perceived as the basis for other values to be perceived. The value of other dimensions can only be discussed if the primary care is functional. Therefore, while strengthening the perception of the emotional value of medical services, we should not look down on the basic diagnosis and treatment functions of grassroots medical institutions.

## Suggestions

To further promote the implementation of the GDT system, strengthen the construction of the system of “grassroots first diagnosis and two-way referral” and achieve the goal of effectively promoting grassroots medical treatment. The key is to improve the mass value perception of grassroots medical care and continue to optimize the comprehensive level of primary medical services. Combined with the conclusions of the questionnaire survey, this paper puts forward some suggestions on how to promote medical treatment at the grassroots level. In the process of policy implementation, measures such as strengthening personalized service in medical institutions, strengthening the training of medical staff, and increasing the input of medical resources at the grassroots level should be combined to improve the effectiveness of policy implementation. Specific suggestions are as follows:

(1) Innovating service modes and paying attention to the emotional needs of the masses

Based on the experimental results of the significant impact of emotional value on grassroots medical treatment, grassroots medical institutions should strengthen the construction of personalized services related to the perception of emotional value. In the post-epidemic era, the explosion of personalized service demand has brought new opportunities to primary medical institutions. However, China's huge population and limited medical resources mismatch, leading to the supply and demand of personalized services, which have not been balanced yet. Therefore, on the one hand, grassroots medical institutions should take advantage of their wide distribution to provide convenient and personalized services to the masses. On the other hand, grassroots medical institutions shall pay attention to the emotional demands of the people for deep needs and medical treatment, actively respond to innovative services, such as family doctor services and regular community visiting services, and improve the sense of belonging and trust of the masses. By strengthening the trust of the masses in primary medical treatment, the loyalty of the masses to primary medical treatment shall be enhanced (22) to improve the long-term primary medical treatment rate.

(2) Strengthen personnel training and improve service capacity

At present, the main factors with low positive perception in the emotional value dimension are the initiative of medical staff and the confidence of patients. The key to improving the level of the above two elements lies in the willingness and ability of grassroots medical personnel. Medical institutions shall strengthen the training of grassroots medical personnel and enhance their professional skills and qualities. At the same time, attention shall be paid to talent incentives to improve the initiative of grassroots medical personnel. In addition, more doctors are unwilling to work at the grassroots level because of the narrow promotion space. Because of this problem, government departments shall give grassroots medical staff fair promotion opportunities for professional title evaluation, and grassroots medical institutions shall improve the incentive system, assist doctors in making career planning, acquire talent sense of belonging with humanistic care, and accumulate reserve force for providing better medical services.

(3) Increase the input of grassroots medical resources and enhance the functionality of medical services

The government should increase the input of grassroots medical resources to solve the problem of relatively low satisfaction with instrument completeness and service effectiveness, improve the functionality of primary medical services by allocating medical equipment involved in general medical examinations. While ensuring the convenience of medical treatment, the relevant departments shall strengthen the connection between grassroots medical institutions and gradually build a sound medical network with the help of the internet. Through the network, we can reduce repeated physical examinations, transform fragmented treatment into continuous treatment, provide more efficient services for the masses, and make the perception of functional value grow steadily.

## Data availability statement

The data that support the findings of this study will be made available from the corresponding author upon reasonable request.

## Author contributions

WT contributed to conceptualization, writing of the original draft, describing the proposed framework, and writing of the review. XC contributed to data curation, formal analysis, and wrote the whole manuscript. SG contributed to data collection, processing, and analysis. All authors contributed to the article and approved the submitted version.

## Funding

This work was supported by the Special Funds for Basic Business Expenses of Colleges and Universities in Zhejiang Province (XT202113), the Xinmiao Talent Program Project in Zhejiang Province (2021R408066), the Zhejiang Provincial Natural Science Foundation of China (LQ22F020001), and the Scientific Research Fund of Zhejiang Provincial Education Department (Y202147355).

## Conflict of interest

Author WT was employed by company Zhejiang Geely Holding Group Co., Ltd. The remaining authors declare that the research was conducted in the absence of any commercial or financial relationships that could be construed as a potential conflict of interest.

## Publisher's note

All claims expressed in this article are solely those of the authors and do not necessarily represent those of their affiliated organizations, or those of the publisher, the editors and the reviewers. Any product that may be evaluated in this article, or claim that may be made by its manufacturer, is not guaranteed or endorsed by the publisher.
